# AI-based derivation of atrial fibrillation phenotypes in the general and critical care populations

**DOI:** 10.1016/j.ebiom.2024.105280

**Published:** 2024-08-16

**Authors:** Ryan A.A. Bellfield, Ivan Olier, Robyn Lotto, Ian Jones, Ellen A. Dawson, Guowei Li, Anil M. Tuladhar, Gregory Y.H. Lip, Sandra Ortega-Martorell

**Affiliations:** a Data Science Research Centre, Liverpool John Moores University, Liverpool L3 3AF, UK; b Liverpool Centre for Cardiovascular Science at University of Liverpool, Liverpool John Moores University and Liverpool Heart & Chest Hospital, Liverpool, UK; c School of Nursing and Advanced Practice, Liverpool John Moores University, Liverpool L2 2ER, UK; d Research Institute for Sport and Exercise Science, Liverpool John Moores University, Liverpool L3 3AF, UK; e Center for Clinical Epidemiology and Methodology (CCEM), Guangdong Second Provincial General Hospital, Guangzhou 510317, China; f Department of Neurology, Radboud University Medical Centre, Donders Institute for Brain, Cognition and Behavior, Nijmegen, the Netherlands; g Danish Center for Health Services Research, Department of Clinical Medicine, Aalborg University, Aalborg, Denmark

**Keywords:** Clinical phenotypes, Atrial fibrillation, Generative topographic mapping, UK-Biobank, MIMIC-IV, Probabilistic modelling, Machine learning, Clustering, Stratification

## Abstract

**Background:**

Atrial fibrillation (AF) is the most common heart arrhythmia worldwide and is linked to a higher risk of mortality and morbidity. To predict AF and AF-related complications, clinical risk scores are commonly employed, but their predictive accuracy is generally limited, given the inherent complexity and heterogeneity of patients with AF. By classifying different presentations of AF into coherent and manageable clinical phenotypes, the development of tailored prevention and treatment strategies can be facilitated. In this study, we propose an artificial intelligence (AI)-based methodology to derive meaningful clinical phenotypes of AF in the general and critical care populations.

**Methods:**

Our approach employs generative topographic mapping, a probabilistic machine learning method, to identify micro-clusters of patients with similar characteristics. It then identifies macro-cluster regions (clinical phenotypes) in the latent space using Ward’s minimum variance method. We applied it to two large cohort databases (UK-Biobank and MIMIC-IV) representing general and critical care populations.

**Findings:**

The proposed methodology showed its ability to derive meaningful clinical phenotypes of AF. Because of its probabilistic foundations, it can enhance the robustness of patient stratification. It also produced interpretable visualisation of complex high-dimensional data, enhancing understanding of the derived phenotypes and their key characteristics. Using our methodology, we identified and characterised clinical phenotypes of AF across diverse patient populations.

**Interpretation:**

Our methodology is robust to noise, can uncover hidden patterns and subgroups, and can elucidate more specific patient profiles, contributing to more robust patient stratification, which could facilitate the tailoring of prevention and treatment programs specific to each phenotype. It can also be applied to other datasets to derive clinically meaningful phenotypes of other conditions.

**Funding:**

This study was funded by the DECIPHER project (LJMU QR-PSF) and the EU project TARGET (10113624).

**Research in context**

**Evidence before this study**

Clinical complexity associated with atrial fibrillation (AF) patients has major implications for treatments and outcomes. To predict AF and AF-related complications, clinical risk scores are commonly employed, but their predictive accuracy is generally limited, given the inherent complexity and heterogeneity of patients with AF. Conventional classification of patients with AF based solely on disease subtypes or arrhythmia patterns (e.g., paroxysmal, persistent, or permanent) may fall short of adequately characterising this diverse population. By classifying different presentations of AF into coherent and manageable clinical phenotypes, the development of tailored prevention and treatment strategies can be facilitated. Previous studies have demonstrated the value of phenotyping, with each identifying between three and six clinically distinct AF phenogroups. However, the methodological approaches followed to derive such phenotypes may not be particularly suited to model complex relationships in the data, and they lack resiliency to data uncertainty and robustness across datasets.

**Added value of this study**

Our study proposes an AI-based probabilistic approach to identify clinically relevant AF phenotypes for specific patient cohorts, from the general and the critical care populations. Our approach can handle uncertainty, is robust to noise, derives more specific patient profiles, and can uncover hidden subgroups, contributing to more robust patient stratification. We tested our methodology on two large databases, and generated phenotypes using two different AF cohorts: one derived from general population data from the UK-Biobank, and the other derived from critically ill patients admitted to the intensive care unit from the MIMIC-IV database. The phenotypes in both cohorts were derived from vitals and laboratory test data (no medical history/comorbidities or demographic data was explicitly included in the modelling stage to prevent possible bias), and remarkably, the derived phenogroups were still able to identify significant differences in those variables when studied post-hoc. Link to the code: (https://zenodo.org/doi/10.5281/zenodo.12207621).

**Implications of all the available evidence**

Using our methodology, we identified and characterised clinical phenotypes of AF across diverse patient populations, which could facilitate the tailoring of prevention and treatment programs specific to each phenotype. The proposed approach not only can be used to extract AF phenotypes but can also be applied to other datasets to derive clinically meaningful phenotypes of other conditions.

## Introduction

Atrial fibrillation (AF) is the commonest heart arrhythmia worldwide,^1^ affecting 2% of the European population (15 M patients). AF risk increases with age, with ∼18 M patients with AF estimated by 2060.^2^ AF is linked to a higher risk of mortality and morbidity from stroke, heart failure, dementia, and hospitalisations. Patients with AF are often associated with various cardiovascular and non-cardiovascular risk factors,^2^ and these often do not occur in isolation, co-existing in clusters of comorbidities, leading to multimorbidity, polypharmacy and frailty.^3^ Such clinical complexity associated with patients with AF major implications for treatments and outcomes.^4^ To predict AF and AF-related complications, clinical risk scores are commonly employed, but their predictive accuracy is generally limited, given the inherent complexity and heterogeneity of patients with AF.

Artificial Intelligence (AI), and more specifically machine learning (ML), is increasingly used in clinical practice for disease prediction and detection, as well as events and treatment optimisation.^5^ Most ML applications in AF leverage supervised ML learning (requiring labelled data), however in recent years, there has been a rise in the application of unsupervised ML approaches as they can be used for exploring and understanding the inherent structure and characteristics of the data without requiring labelled outcomes or targets.

Conventional classification of patients with AF based solely on disease subtypes or arrhythmia patterns (e.g., paroxysmal, persistent, or permanent) may fall short of adequately characterising this diverse population.^1^ The task of categorising patients into meaningful subgroups/phenotypes is inherently challenging and susceptible to misclassification. These phenotypes, in the context of medical research, are constructs based on clinical and physiological measurements that enable the characterisation of patient subgroups within a specific disease.^6^ They comprise either individual disease attributes or combinations thereof, offering a comprehensive description of distinctions among affected individuals, including clinically significant outcomes such as symptoms, exacerbations, treatment responses, disease progression rate, or mortality. By classifying different presentations of AF into coherent and manageable clinical phenotypes, the development of tailored prevention and treatment strategies can be facilitated. This is aligned with the current holistic approach to AF management,^7^ as recommended in guidelines.^8^

Different approaches have been followed previously to identify AF phenotypes such as hierarchal clustering (namely Ward’s minimum variance method^9^, ^10^, ^11^ and complete linkage using Gowers distance^12^) and k-prototype.^1^ These methods are not particularly suited to model complex relationships in the data, they assume clusters are generally homogeneous, they tend to be less interpretable,^13^ they may be sensitive to initialisation,^14^^,^^15^ they may not handle cluster membership uncertainty, and they lack robustness across datasets.^14^ However, these studies all demonstrate the potential value of phenotyping, with each identifying between three and six clinically distinct AF phenogroups. The population groups studied also vary, including Japanese,^1^^,^^10^^,^^16^ European,^9^^,^^11^^,^^17^ and North American^9^ populations.

This study proposes a methodological approach for generating clinically relevant AF phenotypes for specific patient cohorts, from the general and the critical care populations. To test the proposed approach, we generated phenotypes using two different AF cohorts: one derived from general population data from the UK-Biobank, and the other derived from critically ill patients admitted to the intensive care unit (ICU) from the MIMIC-IV database. These databases were chosen as they are both large and offer a rich pool of variables.

Our approach employs generative topographic mapping (GTM),^18^^,^^19^ a probabilistic ML method chosen for its ability to elucidate meaningful data representations from large datasets. AF phenotypes were derived from the GTM model, and the inherent clinical characteristics associated with each of them were explored for both cohorts.

## Methods

### Proposed AI-based methodology to generate reliable phenotypes

#### Micro-cluster segmentation using GTM

**Fig. 1:**
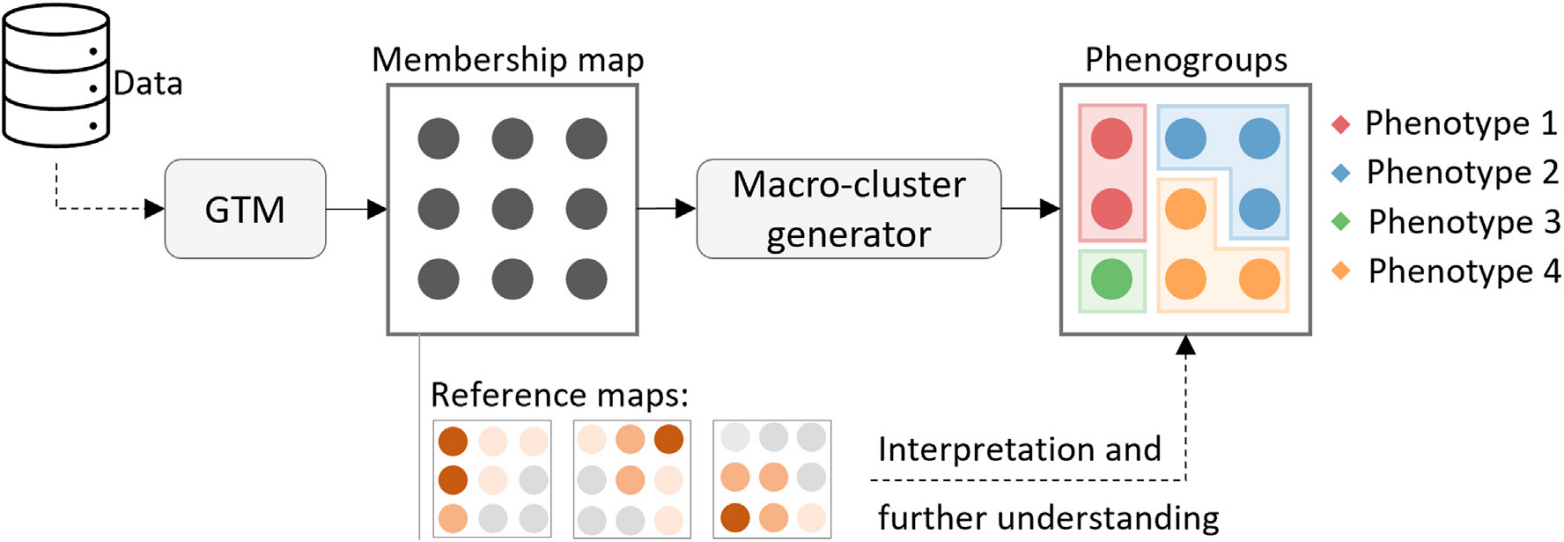
**Proposed AI-based methodology to generate reliable phenotypes.** Data is modelled by the GTM algorithm, which projects the data into a 2-dimensional latent space, visualised in the membership map. The GTM also produces reference maps, which are used to indicate the influence of a variable over a micro-cluster. Hierarchical clustering is then applied to the reference vectors to group similar micro-clusters together into larger macro-clusters, which in turn are used to derive the phenotypes.

Our approach (Fig. 1) first uses GTM, an unsupervised ML methodology grounded in probability theory^18^ that offers a principled alternative to the widely used Self Organising Map algorithm.^20^ GTM addresses several known issues associated with SOM, such as non-guaranteed convergence, limited neighbourhood preservation, lack of an objective function, and the absence of an explicitly defined probability density function.^21^ Alternative algorithms such as t-SNE^22^ and UMAP^23^ have become popular for reducing dimensionality and visualising data. Whilst they have different mathematical underpinnings, both methods aim to reflect the underlying structure of the data. However, as opposed to GTM, they are not probabilistic methods; t-SNE and UMAP are deterministic techniques that focus on preserving local and global structures without explicitly modelling probability distributions. This is a limitation of the latter two methods since we are interested in generating probabilistic representations and explicit cluster modelling for the AF phenotypes. A probabilistic approach would offer advantages such as uncertainty quantification, robustness to noise, more specific patient profiles, and the ability to uncover hidden subgroups, ultimately contributing to a more robust stratification of patients.

GTM operates by assuming first that the observed data are generated through a nonlinear, topology-preserving mapping from a low-dimensional latent space to a high-dimensional data space. Let the data in the original data space D be represented as $x=\left(x_{1},x_{2},\ldots ,x_{D}\right)$ and the latent variables be represented as $u=\left(u_{1},u_{2},\ldots ,u_{L}\right)$. The projection of points from the latent space to the data space is carried out using the non-linear function y(u;W) where W represents a set of parameters that maps points u in the latent space into the points y(u;W) that lie in the data space. The probability density function of the latent space, p(u), is set to the sum of delta functions, as described in eq. (1), constraining the latent points to a uniform discrete grid of centres.

$$\begin{array}{c} p\left(u\right)=\frac{1}{K}\sum_{l=1}^{K}\delta \left(u-u_{l}\right) \end{array}$$

Each centre in the latent space, $x_{l}$, is responsible for generating a spherical Gaussian density function in the data space centred on $y\left(x_{l};W\right)$, with variance β for a given $x_{l}$ and W. The distribution in the dataspace can therefore be understood as a Gaussian mixture model defined by eq. (2).

$$\begin{array}{c} p\left(x|W,\beta \right)=\frac{1}{K}\sum_{l=1}^{K}p\left(x|u_{l},W,\beta \right) \end{array}$$

Where the parameters W and β can be determined by using maximum likelihood, whereby the log-likelihood is defined as

$$\begin{array}{c} L\left(W,\beta \right)=\sum_{n=1}^{N}\ln\left\{\frac{1}{K}\sum_{l=1}^{K}p\left(x_{n}|u_{l},W,\beta \right)\;\right\} \end{array}$$

The optimisation of this log-likelihood is carried out using a variant of the expectation-maximisation (EM) algorithm. For the full details on the calculations, please refer to the original publications.^18^^,^^21^

In practice, GTM calculates the probability of an observed data point, represented in here by a patient/participant, belonging to each cluster. The cluster with the highest probability determines the final cluster assignment, resulting in a fine-grained, micro-segmentation of the original data space. This means that GTM performs soft assignments of patients to clusters. This soft assignment strategy yields data clusters within the latent space, where all participants within a given cluster exhibit similar characteristics. This robust approach minimises the likelihood of data clusters comprising dissimilar participants. Since we have chosen a 2-dimensional latent space, these data clusters can be visually represented on a 2-dimensional map, which we will refer to as the “membership map”.

To perform the GTM modelling, we used the “ugtm” Python package. As with any ML modelling, a crucial step in the development of ML models is the careful selection of appropriate hyperparameters. This is to ensure the model can learn the key relationships within the data whilst minimising the risk of overfitting and ensuring the model can generalise to unseen data. Although there are scenarios where hyperparameter tuning may be less critical with the GTM method, in this context, where the intended use of phenotypes is not purely prescriptive, the paramount objective was to ensure that the model could generalise effectively, and accurately project new, unseen patients into the most fitting phenotype.

Consequently, we conducted a comprehensive search of a predefined parameter space to identify the most suitable hyperparameters for our model. The specific hyperparameters subjected to tuning included the number of latent clusters (and by extension, the number of Gaussian centres in the data space), the number of radial basis functions (RBFs) (denoted as “W") employed for projecting data from the latent space to the data space, and the penalisation term used to regulate the mapping process.

Each combination of hyperparameters underwent rigorous evaluation through 10-fold cross-validation. The primary performance metric for each test involved assessing the negative log-likelihood of the test data fold projections. The optimal hyperparameters were selected based on their ability to perform exceptionally well on the test data while also exhibiting minimal standard deviation across all results from each cross-validation fold. The results of the hyperparameter tuning showed that the parameter set of a latent space grid size of 15 × 15, 196 RBFs arranged in a 14 × 14 grid with a regularisation term of 1 was optimal and was therefore used when training the GTM models for both the UK Biobank and MIMIC-IV cohorts.

After obtaining a trained GTM model, each cluster centre can be seen as a composite representation of the data residing in the observed data space, hereafter referred to as the “reference vector”. The components of these reference vectors, derived from the data used to train the model, serve as the basis for creating reference maps for the variables (Fig. 1), which help to show their influence on each patient cluster through heatmap visualisations, i.e., the intensity of high and low values represents the extent to which each variable influences different areas of the membership map. An additional approach to interpreting the clusters involves superimposing other variables not seen by the model during the training, presented in the form of a heatmap onto the membership map visualisations. This provides users with an alternative method for comprehending the clusters through post-hoc analysis.

A crucial property of GTM is the preservation of data topology, meaning that similar clusters should be positioned closer together in the latent space. Even if the most probable cluster assigned to a participant does not precisely correspond to the actual one, it is expected to be closer to the correct one. This makes GTM representations valuable for visualising complex high-dimensional data in a more interpretable lower-dimensional space. In contrast, common clustering techniques such as k-means, lacking probabilistic foundations, are not specifically designed to handle such levels of uncertainty.

#### Macro-cluster analysis to generate AF phenotypes

Defining macro-clusters within the array of micro-clusters generated by GTM is crucial for the identification of AF phenotypes. The outcome of such analysis would shed light on regions in the latent space where micro-clusters with similar characteristics are concentrated, representing natural groupings and inherent common patterns in the data space. As defined in eq. (2), the centres in the latent space are projected into the data space to create a non-linear manifold using GTM.

Our approach (Fig. 1) was inspired by an algorithm introduced by Vellido et al.^24^ Instead of identifying macro-cluster regions in the latent space, we used agglomerative hierarchal clustering using Ward’s minimum variance method^25^ on the reference vectors, and the distances between the vectors were computed using the Euclidean metric. The reference vectors corresponded to the Gaussian centres projected from the centres in the latent space, each residing in the data space. Subsequently, the cluster assignment of each reference vector was mapped to their respective centres in the latent space, effectively generating the desired macro-clusters comprising the latent space’s micro-cluster centres. The full code implementing this approach can be found on Zenodo^26^ at the following link (https://zenodo.org/doi/10.5281/zenodo.12207621).

### Data used for deriving AF phenotypes

#### Modelling variables extracted from the UK-Biobank database

The first dataset used for this analysis was a subset extracted from the UK-Biobank, a large, population-based database^27^ encompassing over 500,000 participants aged 40–69 from across the UK. To identify eligible participants with AF, we searched ICD-10 codes related to AF diagnosis recorded in the participants’ conditions and causes of death variables. Eligible participants would have at least one of these codes recorded. See list of codes in SM, Supplementary Table S1.

In total, 67 variables from the UK-Biobank were used for modelling, 40 genomic variables and 27 biological sample variables. We only included these variables to ensure that participants were clustered based on the similarity of their biological and genetic profiles, rather than being influenced by external demographic factors. The genomic variables are a set of 40 principal components generated using >100,000 single nucleotide polymorphisms (SNPs).^28^ The 27 biological sample variables selected aim to represent key risk markers associated with AF: clotting, inflammation, renal function, liver function, cholesterol, diabetes, and sex-related markers.^29^

#### Modelling variables extracted from the MIMIC-IV database

Data was extracted from the Medical Information Mart for Intensive Care IV (MIMIC-IV^30^), a freely available database of de-identified electronic health records linked to patients admitted to the Beth Israel Deaconess Medical Centre in Boston, Massachusetts. We used version 2.2 (January/2023), which includes 73,181 ICU stays.

Patients were included in this study if they had at least one episode of AF during the ICU admission. The latter was extracted from the *chartevent* table, using the code for heart rhythm: 220048, and identifying from those the ones that have value “AF (Atrial Fibrillation)”. Therefore, this would include patients with pre-existing AF, and those with new-onset AF, although the first AF episode recorded occurred after the first 24 h of the ICU admission. Patients <18 years old, patient admissions with short ICU stays (<24 h), and patients with multiple ICU stays were excluded from the study.

In total, 21 variables from the MIMIC-IV database were used for modelling. These variables were extracted from sequences of vitals (e.g., temperature, and heart rate) and lab test results (e.g., glucose and haemoglobin) used to monitor the condition of the patient in the ICU. The variables used for modelling were selected as they represent key risk markers associated with AF in ICU.^31^^,^^32^

### Selection of variables associated with AF

#### AF in the general population: UK-Biobank data

AF is associated with ageing and comorbidities, as reflected in our phenotypic data. Indeed, multiple studies have shown how comorbid risk factors do not occur in isolation, but cluster together contributing to clinical complexity phenotypes.^3^^,^^4^ There are well-recognised associations of common comorbidities such as hypertension, heart failure and diabetes, as well as renal and liver dysfunction.^33^ The choice of biological sample variables selected for our modelling aims to represent key risk markers associated with AF since they are essential for a comprehensive understanding of the factors contributing to AF. For example, inflammatory processes play a role in the development and progression of AF.^34^ Certain genetic variants have also shown significant association with silent AF.^35^

Various risk prediction tools have been proposed for the prediction of incident AF,^36^ e.g., CHARGE-AF (The Cohorts for Heart and Ageing Research in Genomic Epidemiology AF) score, developed for the general population, which uses variables such as age, ethnicity, height, weight, blood pressure, medication use, and comorbidities.^37^ Simpler clinical risk factor scores such as C_2_HEST have also been investigated to predict incident AF in population and post-stroke cohorts.^38^

#### AF in the critical care population: UK-Biobank data

AF stands as the most prevalent arrhythmia among critically ill patients, occurring at an incidence rate of 10–15%^39^ within the critical care population. Patients in the ICU that have AF suffer with a worse prognosis, longer ICU stays and higher mortality.^40^ Treatments for managing AF that are used for patients in the general population may not be appropriate for critically ill patients,^41^ therefore having ICU focused results is crucial for optimising patient outcomes. The risk factors for AF can significantly differ between the general and the critical care populations. Common risk factors for AF in the community involve structural and valvular heart disease, but these factors may not be distinctly associated with AF in critical illness.^42^ In addition, acute factors are thought to be associated with increased risk for newly diagnosed AF during critical illness.^37^ For example, invasive ventilation is associated with AF episodes in critically ill patients.^42^ Monitoring oxygenation is crucial in these patients to assess respiratory function and optimise oxygen delivery, as compromised oxygenation can exacerbate cardiovascular stress and contribute to complications.^43^ Electrolyte imbalances, such as phosphate abnormalities, observed in medical conditions like kidney dysfunction, may indirectly contribute to AF development.

### Additional investigative variables

Additional investigative variables were extracted for further exploration, and they were not used during model development. Instead, they are employed post-hoc to provide further context/insights related to the composition of individual or group of clusters and to help identify potential meaningful AF phenotypes.

#### Investigative variables extracted from the UK-Biobank database

We used a set of 18 UK-Biobank variables for visualisation purposes. This selection consisted of 15 assessment centre variables, and two population characteristic variables, with the remaining variable belonging to the health-related outcomes category. Several of these variables were previously identified in prior AF studies^29^ and includes sex (determined either from the NHS central registry or by what was self-reported by the participant), BMI, activity levels and alcohol consumption.

We consider that incorporating comorbidity data is fundamental for understanding how various medical conditions can be differentiated among clusters of AF participants in the general population. To effectively convey information on thousands of diverse comorbidities in a clear, meaningful manner, we integrated the use of *phecodes*.^44^ Each phecode is composed of several individual diagnoses, defined using ICD-10 codes, which are subsequently grouped into various phecode categories.

In our analysis of AF participants from the general population using UK-Biobank data, we included several phecode categories that encompassed diagnoses from a predefined set of comorbidities commonly associated with individuals suffering from AF. To assign a phecode, and subsequently associate it with a phecode category, a patient’s record was examined for a match with the ICD-10 code of either primary or secondary diagnoses to one within a phecode. The list of all phecodes, and their respective phecode categories, that were considered in this study can be found in Supplementary Table S2. For the full details regarding which ICD10 codes make up each phecode, please refer to the original publication.^44^

#### Investigative variables extracted from the MIMIC-IV database

A selection of 27 variables from the MIMIC-IV database were extracted for further investigation. They include demographic data such as sex (reported in the dataset as gender however we used this term as it is more appropriate as it refers to the biological sex of the patient), age, and ethnicity. They also include the Glasgow Coma Scale (GCS), a neurological assessment tool commonly employed in critical care settings, which is used to evaluate a patient’s level of consciousness based on their eye, verbal, and motor responses. Ventilation status (invasive and non-invasive), acute kidney injury (AKI) and acute respiratory distress syndrome (ARDS) are also investigated as variables of interest, as well as a series of variables related to length of stay and mortality.

### Data pre-processing

To ensure the development of a robust and representative dataset for modelling, we undertook several pre-processing steps. First, we implemented a set of missingness criteria (defining appropriate levels/thresholds of data completion) to determine which variables and participants to include. The thresholds were set at 25% and 30% for data that could be missing for a variable or a participant, respectively. We also identified certain variables that exhibited positive skewness in their value distributions. To address this, we applied a log transformation to these variables, rendering their distributions more Gaussian in nature.

Subsequently, any remaining missing data were addressed through imputation, employing a multivariate imputer. This imputer estimated missing values by considering known values from other variables. To accomplish this, we utilised the “IterativeImputer” function, which is part of the Scikit-Learn Python package and draws inspiration from the R MICE package 6. Invalid values of the variables (e.g., heart rate < 0) were marked as not available. Variables recorded with different units were harmonised, e.g., in MIMIC-IV, height was present in inches and centimetres (cm), and they were all converted to cm.

### Ethics

The UK Biobank is approved from the North West Multi-centre Research Ethics Committee as a Research Tissue Bank and researchers do not require separate ethical clearance. The use of MIMIC-IV data did not require ethical approval as the analysis is based on secondary data which is publicly available, and no permission is required to access the data.

### Statistics

Medians and interquartile ranges were calculated for continuous variables, and frequencies and proportions (percentages) were used for categorical variables. There were several ordinal variables used for the exploratory analysis of the GTM output. These were one-hot encoded and then treated as a categorical variable and represented in the data as such.

To study the characteristics of the generated phenotype groups, differences between continuous variables were analysed using the Kruskal–Wallis test and differences between categorical variables were analysed using the Chi-squared test. In both cases, a p-value < 0.05 was the threshold for statistical significance.

### Role of funders

The funders did not participate in the study’s design and implementation, data collection, management, analysis, or interpretation. They were also not involved in the preparation, review, or approval of the manuscript, nor in the decision to submit it for publication.

## Results

### Characteristics of the participants/patients cohorts

From the UK-Biobank we extracted 36,680 participants with AF from this general population cohort using the criteria set out in 2.2.1 (median age 63 years (IQR 59–67), range 40–72 years; 63.5% male). Table 1 contains the summary of the biological variables used for modelling, and the investigative variables used in the post-hoc analysis. A second dataset of 2695 critically ill patients with AF using the criteria set out in section 2.2.2 (median age 73 years (IQR 65–81), range 21–89 years; 60.4% male) was extracted from the MIMIC-IV (Table 2).

**Table 1:** Characteristics of the participant subset extracted from the UK-Biobank database.




All data presented below was taken from the first data instance available. Medians and interquartile ranges were calculated for continuous variables, and frequencies and proportions (as percentages) were calculated for the categorical variables. Red shades were used for the modelling variables, whilst blue was used for the additional investigative variables.

**Table 2:** Characteristics of the ICU patient subset extracted from the MIMIC-IV database.

Summary statistics and colours as in Table 1. The data represented for each variable is the average of all data recorded during the ICU stay.

### Visualisation of reference vectors for the modelling variables

#### Reference vectors of the modelling variables – used to derive AF phenotypes

**Fig. 2:**
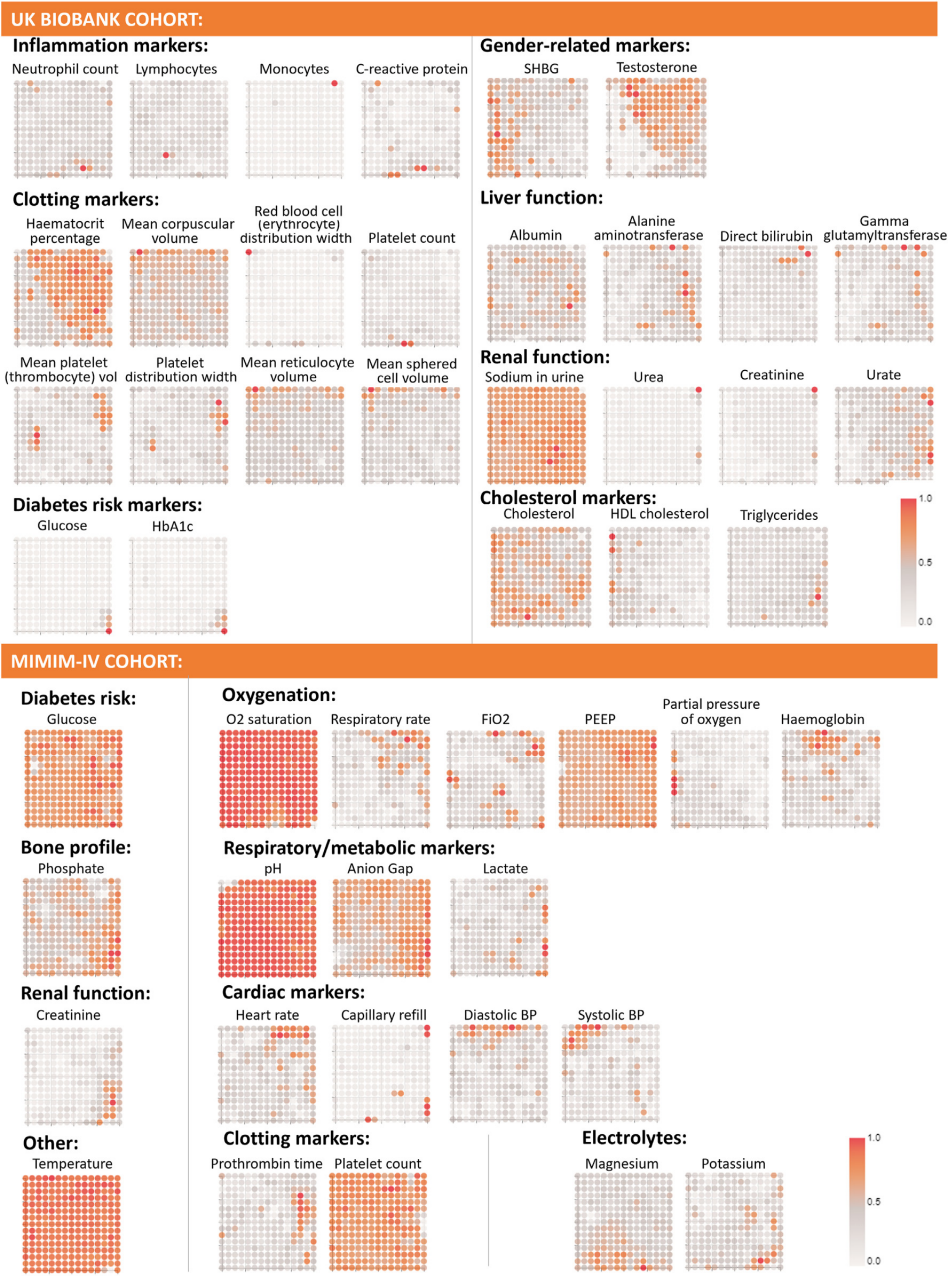
**Reference vector visualisations demonstrating how each biological sample variable affects the cluster distribution in the latent space for both, the UK-Biobank and the MIMIC-IV AF cohorts**.

Fig. 2 contains the reference vectors extracted from the trained GTM models for the UK-Biobank and MIMIC-IV AF cohorts. For the UK-Biobank data, it contains the reference vectors for the biological sample variables, with plots grouped by the different risk factors they relate to, whilst for the MIMIC-IV, it displays all modelling variables used for modelling. Each point in every plot within Fig. 2 corresponds exactly to the same point in their respective membership map (SM, Supplementary Fig. S1). A light grey–red colour scheme was used for the reference vectors plot such that areas of the plots that are redder indicate that participants in that cluster had a higher value of that variable. Likewise, if the point in the reference vector is greyer, the lower the value is for participants in this cluster. All plots using the light grey–red colour scheme indicate variables used in the GTM model development, whereas plots using a light grey–teal represent variables that were not used in the modelling and have no direct impact on the clusters themselves.

#### Visualisation of the additional investigative variables

**Fig. 3:**
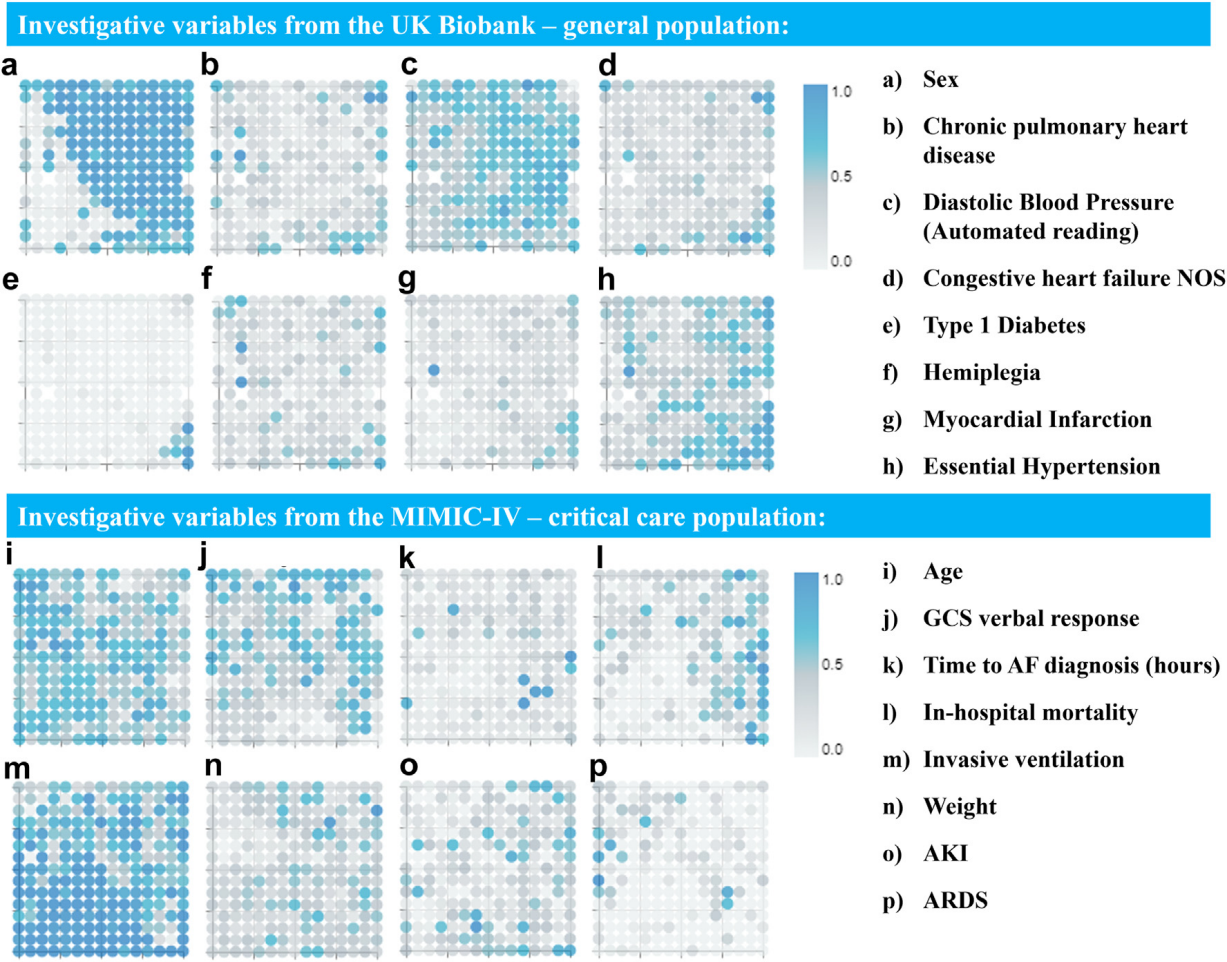
**Membership maps showing how a selection of investigative variables data are distributed within the latent space for the UK-Biobank and the MIMIC-IV cohorts.** AF: Atrial Fibrillation. AKI: Acute Kidney Injury. ARDS: Acute Respiratory Distress Syndrome. GCS: Glasgow Coma Scale.

Fig. 3 contains a selection of visualisations showing how data from different investigative variables are distributed within the membership maps for the UK-Biobank and MIMIC-IV cohorts. The visualisations representing the investigative variables all use a light grey-teal colour scheme as they were not used in model development. The value assigned to each micro-cluster is the average of the variable for all participants assigned to each cluster, the more teal a micro-cluster is, the higher the value. In SM, section 5, visualisations for all investigated variables are displayed.

### Description of AF phenotypes

**Fig. 4:**
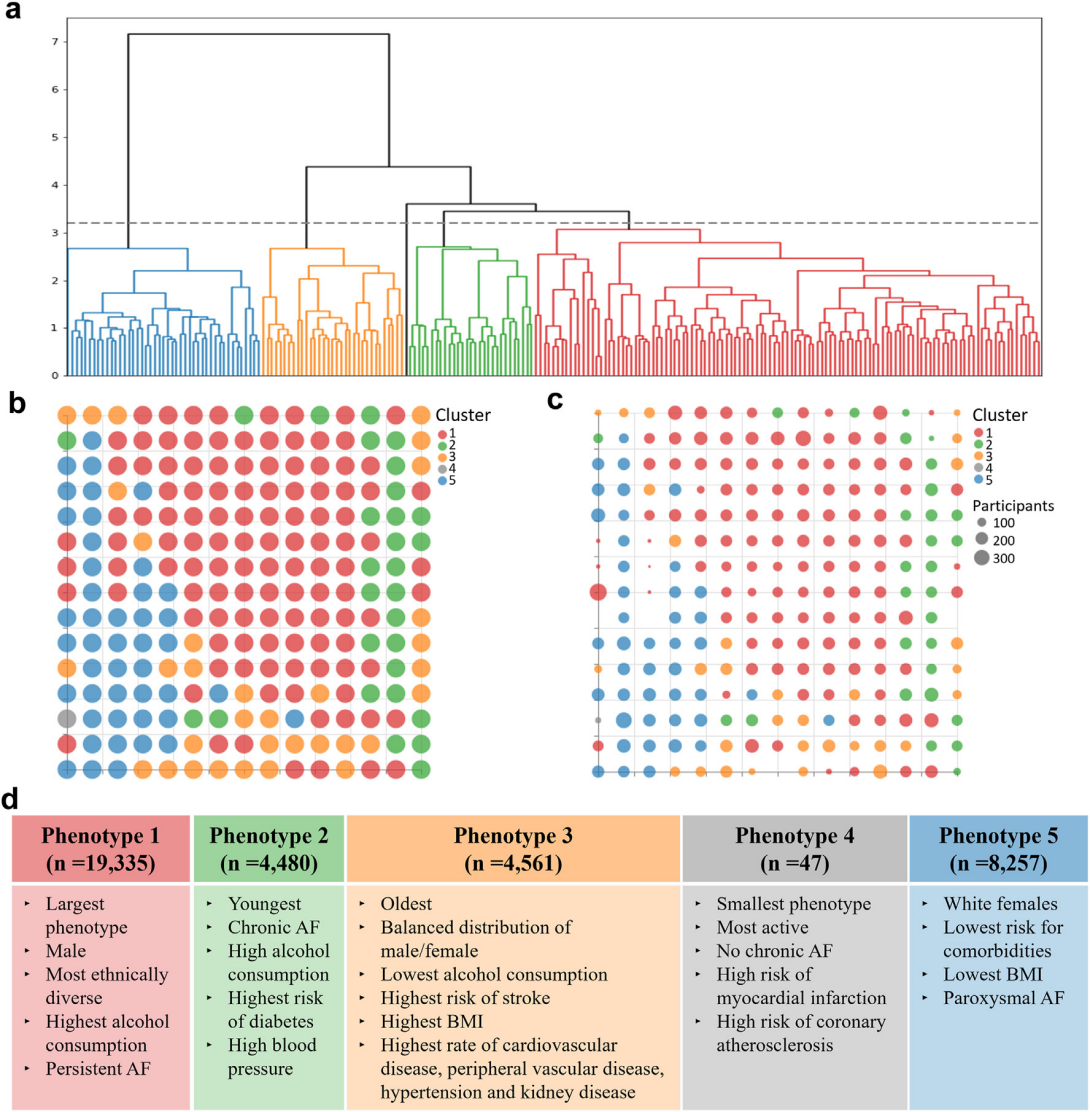
**Derived phenotypes of AF in the general population using UK-Biobank data.** a) Dendrogram produced using Ward’s minimum variance method. The graph shows the 5 clusters that are used to define the 5 AF phenotypes for the general population. b) Membership map with a uniform size for the micro-clusters to show the distribution of the macro-cluster regions. c) The size of the micro-clusters in the membership map dictated by the number of participants assigned to it. d) Main characterising features for each of the phenotypes.

For the UK-Biobank cohort, we identified five clusters within the reference vectors residing in the data space, as demonstrated by the dendrogram in Fig. 4(a). Transferring these reference vector cluster assignments to their corresponding latent centres gave five macro-cluster regions, which in turn were used to define the five AF phenotypes. These macro-cluster regions are visualised in Fig. 4(b) and (c).

**Fig. 5:**
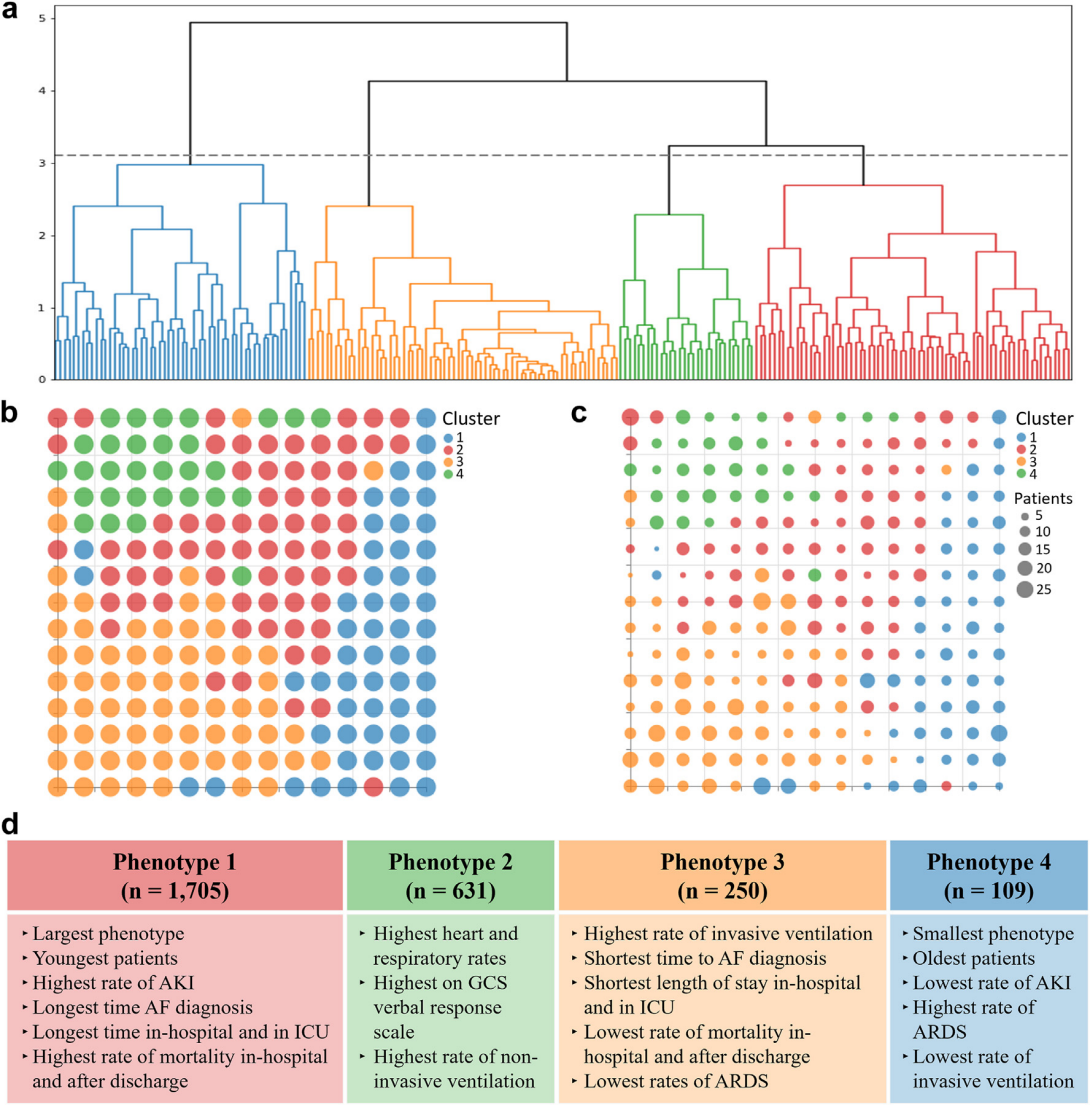
**Derived phenotypes of AF in the general population using MIMIC-IV data.** a) Dendrogram produced using Ward’s minimum variance method. The graph shows the 4 clusters that are used to define the 4 AF phenotypes for ICU patients. b) Membership map with a uniform size for the micro-clusters to show the distribution of the macro-cluster regions. c) The size of the micro-clusters in the membership map dictated by the number of participants assigned to it. d) Main characterising features for each of the phenotypes.

When applied to the MIMIC-IV cohort, the analysis identified four clusters within the reference vectors, as presented in Fig. 5(a). The macro-cluster regions generated by transferring these clusters to their respective latent centres are presented in Fig. 5(b) and (c). The baseline data for each of the two databases were split according to the number of phenotypes and compared, in Table 3, Table 4 for the UK-Biobank and MIMIC-IV data, respectively. A description of the headline features that characterise both sets of phenotypes can be found in Fig. 4, Fig. 5.

**Table 3:** Characteristics of the participants per phenotype of AF in the general population using UK-Biobank data.






As in Table 1, medians and interquartile ranges were calculated for continuous variables, and frequencies and proportions (as percentages) were calculated for the categorical variables. Shades of red/blue were used per variable to illustrate differences between lower and higher values. Red shades were used for the modelling variables, whilst blue was used for the additional investigative variables.

**Table 4:** Characteristics of the participants per phenotype of AF in an ICU population using the MIMIC-IV database.




As in Table 2, medians and interquartile ranges were calculated for continuous variables, and frequencies and proportions (as percentages) were calculated for the categorical variables. As in Table 3, shades of red/blue were used per variable to illustrate differences between lower and higher values. Red shades were used for the modelling variables, whilst blue was used for the additional investigative variables.

### Interpreting the visualisations

**Fig. 6:**
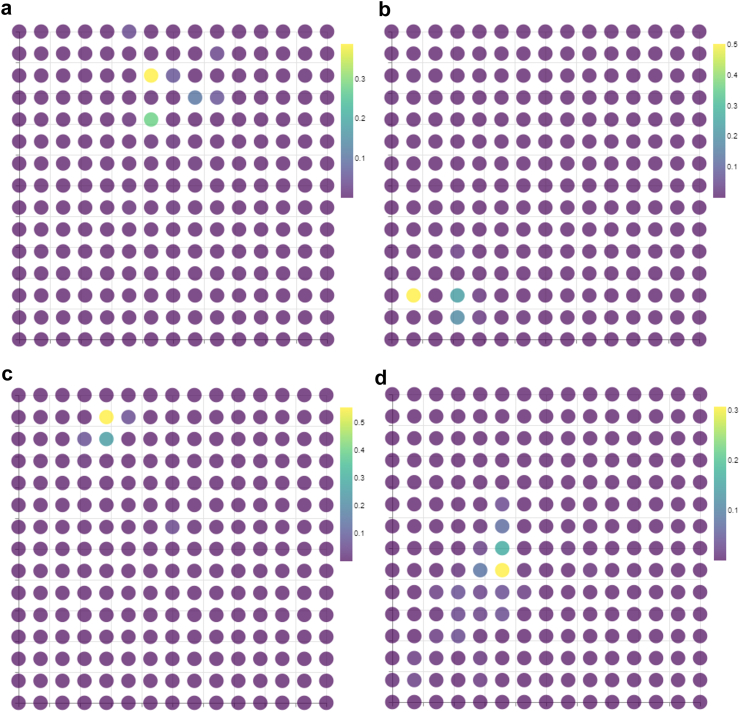
**Membership map with the probability distributions for different data points superimposed.** Maps a) and b) show the probability distribution for two randomly selected participants from the general population taken from the UK Biobank database. Maps c) and d) show the probability distribution for two randomly selected patients from the critical care population taken from the MIMIC-IV database.

The membership maps show us which participants share the same cluster indicating that they share similar features. The probabilistic foundations of GTM allows us to calculate, for each data point, the probability that it was generated from the ith latent node. By calculating the probability for each latent node and overlaying the result onto the membership it allows the user to visualise the probability distribution for each data point. Some examples of this are displayed in Fig. 6. Fig. 6(a) and (b) are the probability distributions for participants from the UK Biobank dataset and Fig. 6(c) and (d) are the probability distributions for patients from the MIMIC-IV dataset. As discussed in section 2.1.1, the latent node that has the highest distribution of generating the data point determines its final cluster assignment. These plots illustrate the soft cluster assignments GTM performs, whilst also demonstrating the robustness of the approach in so far as the next highest probability surround the node the data point was assigned to.

To unlock deeper insights, superimposing modelling data onto the membership maps provides a better understanding of why patients were clustered in such a way (Fig. 2, Fig. 3). Extra insights can be learnt by superimposing post-hoc data, unseen during modelling. One example from the UK-Biobank cohort relates to sex-related markers, specifically testosterone and SHBG levels. By assessing their respective reference vectors, individuals with higher testosterone and lower SHBG tended to be in the middle and top-right sections of the membership map. In contrast, those with heightened SHBG and lower testosterone were clustered towards the bottom left. Given that testosterone levels are generally higher in males,^45^ and SHBG levels are typically elevated in females,^46^ we can deduce that the membership map effectively delineated male and female participants during clustering. This can be seen in Fig. 3(A), where we visually represent the participants’ sex (the bluer area in Fig. 3(A) predominantly corresponds to males), and in Supplementary Fig. S2 (in SM), which shows the membership map stratified by sex.

## Discussion

Using our AI methodology, we have identified and characterised clinical phenotypes of AF across diverse patient populations, which could facilitate the tailoring of prevention and treatment programs specific to each phenotype. The principal findings of this study are: (i) The proposed AI-based methodology showed its ability to derive meaningful clinical phenotypes of AF in the general and critical care populations. (ii) Our approach is probabilistic, offering advantages such as the ability to handle uncertainty, robustness to noise, more specific patient profiles, and the ability to uncover hidden subgroups, contributing to more robust patient stratification and visualising complex high-dimensional data in a more interpretable lower-dimensional space, enhancing understanding.

Identifying clinical phenotypes of diseases using methods like hierarchical clustering (specifically Ward’s minimum variance method and complete linkage with Gowers distance) and k-prototype used in previous phenotyping studies,^9^, ^10^, ^11^, ^12^ may not always be the best option for several reasons: (i) Clinical data often contains diverse information, and these methods may not effectively capture the complexity of relationships within the data, and they may also be influenced by outliers or noise.^47^ (ii) In clinical phenotyping, diseases may exhibit considerable heterogeneity,^9^, ^10^, ^11^, ^12^ however hierarchical clustering assumes that data points within a cluster are homogeneous. (iii) High-dimensional clinical data may pose challenges for hierarchical clustering and k-prototype methods for interpreting results, which in the context of clinical phenotypes may render unintuitive.^13^^,^^47^ (iv) In the case of k-prototype, it can be sensitive to the choice of initial cluster centroids and may converge to local minima.^13^ (v) Clinical data often includes a mix of continuous and categorical variables. Some clustering methods, like k-prototype, handle both types, but the integration of different variable types can be challenging and may not fully capture the information. (vi) Results obtained from these methods may not generalise well across different datasets or populations due to variations in data characteristics.^11^ (vii) They lack probabilistic foundations and hence are not specifically designed to handle such levels of uncertainty.^18^^,^^19^

Alternative approaches, such as probabilistic or ensemble methods, may provide more robust and interpretable clinical phenotypes. Our approach involves deriving micro-clusters using a probabilistic method (i.e., GTM), followed by hierarchical clustering to identify macro-clusters, i.e., the phenotypes. The latter differs from previous studies as the hierarchical methods were applied to the reference vectors from a probabilistic model rather than the original data space, which makes the clusters more stable and resilient to data uncertainty. Our use of GTM often provides highly interpretable representations as it explicitly models clusters and prototypes, offering insights into the underlying structure of the data. The membership map produced by GTM captures the underlying relationships and clusters within the data by mapping data points to these prototypes. This enables comprehensible and interpretable representations of complex data, aiding in knowledge extraction and facilitating insights that might otherwise remain hidden in the original high-dimensional space. Indeed, GTM has been applied in diverse real-world situations spanning various domains such as bioinformatics^48^^,^^49^; in the financial sector^50^; and more recently also in modelling freedom of expression.^51^ To the best of our knowledge, GTM has not been used before to study AF or to generate clinical phenotypes.

The identification and characterisation of clinical phenotypes of AF across diverse patient populations show potential for personalised risk assessment and prognosis. Leveraging these phenotypes could facilitate the tailoring of prevention and treatment programs specific to each phenotype.

The proposed methodology provides several advantages to extract meaningful phenotypes. First, as opposed to previous approaches,^1^^,^^9^^,^^10^^,^^12^^,^^17^ we define phenotypes based on a non-linear clustering approach which can capture more complex relationships. Furthermore, we can visualise the clusters, and by extension the phenotypes, and how each variable affects each cluster, which provides interpretability, crucial for validation and understanding. It also allows for a convenient method of looking at phenotype differences. For example, phenotype 2 in Fig. 4(b) occupies predominantly the right side of the membership map. The reference vector for glucose in Fig. 2 (top) highlights that participants in the bottom right micro-clusters have the highest glucose values when compared to the other micro-clusters. This information can be translated back to phenotype 2 to provide more context about its participants, and how risk factors may not be uniformly distributed within a given phenotype.

Comparing the phenotypes of previous studies with those derived from our proposed methodology is not straightforward. Starting with the general population phenotypes generated using the UK Biobank data, the population we analyse (UK) differs from the Japanese,^1^^,^^10^^,^^16^ European,^9^^,^^11^^,^^17^ and North American^9^ populations previously analysed. As determinants of AF can greatly differ across geographical locations,^9^ this introduces a certain level of expected difference between our results and those already stated. However, one example that stands out is that phenotype 2 (Fig. 4(d)) matches almost identically to cluster 3 identified as part the study conducted by Vitolo et al.*,*^9^ which groups the youngest participants/patients who are likely to be male with high burden of cardiovascular comorbidities and risk factors, along with the highest rates of chronic (permanent) AF. We also see other similarities however they are not fully homogeneous, for example comparing phenotype 3 again in Fig. 4(d) with cluster 2 outlined in the study by Bisson et al.^17^ They both group together the oldest patients/participants with a high prevalence of cardiac conditions, however they differ in that phenotype 3 is split between Male and Female, whereas cluster 2 defined in Bisson et al. is mostly male with almost exclusively permanent AF. What this does indicate is that our approach is able to capture the key relationships between patients with AF and find population-specific relationships that allow the phenotypes to be more representative. The phenotypes generated for the critical care population in our study will be inherently different to the general population, which means a comparison with those developed in the literature would not be appropriate.

Another key difference lies in the selection of modelling variables. The phenotypes for both data cohorts were generated using only vitals and laboratory test data, as opposed to previous studies that also included demographics and medical history/comorbidity information in the modelling. This results in their stated phenotypes having significant differences for such variables as they were used to initially stratify the data. The phenotypes generated in our study show significant differences with these key risk factors, but without including explicit information on these variables during modelling. Additionally, as the between-phenotype differences for variables such as demographics and comorbidities are performed post-hoc, should new data become available from variables not yet examined, their distribution between and within each phenotype can be swiftly identified.

From a clinical perspective, the availability of reliable and robust phenotypes could be a major asset to their assortment of diagnostic tools. Phenotypes provide a different way of visualising a targeted population, which for context of this study is patients with AF. Many of these patients have multiple comorbidities, and management based on a single comorbidity in a binary (yes/no) matter is inappropriate, as many comorbidities tend to cluster leading to clinically complex phenotypes. While clustering can be performed using biostatistical approaches, our proposed methodology using GTM provides a more principled approach to clustering, with the capacity to elucidate more specific patient profiles. This would result in more robust patient stratification, as well as the tailoring of prevention and treatment programs specific to each phenotype.

One of the limitations of this study relates to the genomic principal components used for the UK-Biobank cohort, as their loadings were not available, limiting the ability to interpret them. Another limitation is related to the transferability of the derived phenotypic clusters to other cohorts of data, as they could vary across diverse populations due to genetic, environmental, and cultural differences. Additionally, differences in clinical settings, such as healthcare access, diagnostic criteria, and treatment approaches, may contribute to distinct phenotypic patterns among various patient groups. Since this study’s main objective is to present a robust AI methodology for the derivation of AF phenotypes, this limitation can be mitigated by the derivation of specific phenotypes for different patient cohorts, as and when required. The dynamic nature of risk is also another possible limitation, as the current approach does not address how phenotypes change over time.

Our study proposed an AI-based approach for the derivation of clinically meaningful AF phenotypes. We applied it to two large cohort databases representing general and critical care populations. Our approach is probabilistic, contributing to robust patient stratification. It produces interpretable visualisation of complex high-dimensional data, enhancing understanding. It showed its ability to identify clinical phenotypes of AF, which could enable prevention and treatment programs specific to each phenotype. Our methodology can be applied to other datasets to derive clinically meaningful phenotypes of other conditions.

## Contributors

S.O.M. conceptualised the methodological approach and led the study. S.O.M., I.O., G.Y.H.L., R.L., I.J., A.M.T, and E.A.D. secured the funding. R.A.A.B., I.O. and S.O.M. extracted the data. R.L., I.J. and G.Y.H.L. advised on the selection of clinically relevant variables. R.A.A.B. implemented the code and ran the experiments. S.O.M. and I.O. supervised the study. E.A.D., A.M.T. and G.L. contributed to the discussions. R.A.A.B., S.O.M. and I.O. drafted the early version of the manuscript. All authors reviewed and edited the final manuscript. All authors have read and agreed to the published version of the manuscript.

## Data sharing statement

The UK Biobank database is available for approved projects only (application process detailed at https://www.ukbiobank.ac.uk/enable-your-research/apply-for-access) through the UK Biobank Access Management System (https://www.ukbiobank.ac.uk). The MIMIC-IV database is available on the PhysioNet portal (https://physionet.org/content/mimiciv/2.2/) for credentialed users only.

## Declaration of interests

All authors declare no competing interests.
