# Supplementary material for: AI-based derivation of atrial fibrillation phenotypes in the general and critical care populations
Source: eBioMedicine. 2024 Aug 16;107:105280. doi: 10.1016/j.ebiom.2024.105280 (PMC11381622; doi:10.1016/j.ebiom.2024.105280)
Supplement: Supplementary Material [file mmc1.docx]

**AI-based derivation of atrial fibrillation phenotypes in the general and critical care populations**

Supplementary Materials

# **List of codes used to identify eligible AF participants from the UK-Biobank**

**Table S1. Criteria used to identify eligible AF participants from the UK-Biobank**

| Field ID | Variable Value | Field ID - Description | Variable Value - Description |
| --- | --- | --- | --- |
| 20002 | 1471 | Non-cancer illness code, self-reported | atrial fibrillation |
| 20002 | 1483 | Non-cancer illness code, self-reported | atrial flutter |
| 41270 | I48 | Diagnoses - (main/secondary) ICD10 | Atrial fibrillation and flutter |
| 41270 | I480 | Diagnoses - (main/secondary) ICD10 | Paroxysmal atrial fibrillation |
| 41270 | I481 | Diagnoses - (main/secondary) ICD10 | Persistent atrial fibrillation |
| 41270 | I482 | Diagnoses - (main/secondary) ICD10 | Chronic atrial fibrillation |
| 41270 | I483 | Diagnoses - (main/secondary) ICD10 | Typical atrial fibrillation |
| 41270 | I484 | Diagnoses - (main/secondary) ICD10 | Atypical atrial flutter |
| 41270 | I489 | Diagnoses - (main/secondary) ICD10 | Atrial fibrillation and atrial flutter, unspecified |
| 40001 | I48 | Underlying (primary) cause of death: ICD10 | Atrial fibrillation and flutter |
| 40001 | I480 | Underlying (primary) cause of death: ICD10 | Paroxysmal atrial fibrillation |
| 40001 | I489 | Underlying (primary) cause of death: ICD10 | Atrial fibrillation and atrial flutter, unspecified |
| 40002 | I48 | Contributory (secondary) causes of death: ICD10 | Atrial fibrillation and flutter |
| 40002 | I480 | Contributory (secondary) causes of death: ICD10 | Paroxysmal atrial fibrillation |
| 40002 | I482 | Contributory (secondary) causes of death: ICD10 | Chronic atrial fibrillation |
| 40002 | I489 | Contributory (secondary) causes of death: ICD10 | Atrial fibrillation and atrial flutter, unspecified |
| 131350 | * Any Date | Date I48 First reported | Present |

# **Further details of the data used for analysis**

**Table S2. List of phecodes, and their respective phecode categories, used in this study**

| Phecode Category | Phecode |
| --- | --- |
| Circulatory System |  |
|  | Cardiac complications, not elsewhere classified |
|  | Chronic pulmonary heart disease |
|  | Chronic venous hypertension |
|  | Congestive heart failure (CHF) NOS |
|  | Coronary atherosclerosis |
|  | Essential hypertension |
|  | Heart failure NOS |
|  | Heart failure with preserved EF [Diastolic heart failure] |
|  | Heart failure with reduced EF [Systolic or combined heart failure] |
|  | Hypertension |
|  | Late effects of cerebrovascular disease |
|  | Myocardial infarction |
|  | Other forms of chronic heart disease |
|  | Other hypertensive complications |
|  | Other specified peripheral vascular diseases |
|  | Peripheral vascular disease, unspecified |
|  | Primary pulmonary hypertension |
| Dermatologic |  |
|  | Unspecified diffuse connective tissue disease |
| Digestive |  |
|  | Liver abscess and sequelae of chronic liver disease |
|  | Other chronic non-alcoholic liver disease |
|  | Other disorders of liver |
| Endocrine/Metabolic |  |
|  | Acquired hypothyroidism |
|  | Congenital hypothyroidism |
|  | Diabetes insipidus |
|  | Diabetes mellitus |
|  | Diabetes type 1 with peripheral circulatory disorders |
|  | Diabetes type 2 with peripheral circulatory disorders |
|  | Diabetic retinopathy |
|  | Hypothyroidism NOS |
|  | Polyneuropathy in diabetes |
|  | Secondary diabetes mellitus |
|  | Secondary hypothyroidism |
|  | Type 1 diabetes |
|  | Type 1 diabetes with ketoacidosis |
|  | Type 1 diabetes with neurological manifestations |
|  | Type 1 diabetes with ophthalmic manifestations |
|  | Type 1 diabetes with renal manifestations |
|  | Type 2 diabetes |
|  | Type 2 diabetes with ketoacidosis |
|  | Type 2 diabetes with neurological manifestations |
|  | Type 2 diabetes with ophthalmic manifestations |
|  | Type 2 diabetes with renal manifestations |
| Genitourinary |  |
|  | End-stage renal disease |
| Mental Disorders |  |
|  | Alcoholic liver damage |
|  | Alcoholism |
|  | Delirium dementia and amnestic and other cognitive disorders |
|  | Dementia with cerebral degenerations |
|  | Dementias |
|  | Senile dementia |
|  | Vascular dementia |
| Neoplasms |  |
|  | Basal cell carcinoma |
|  | Bone cancer |
|  | Bone marrow or stem cell transplant |
|  | Breast cancer |
|  | Breast cancer [female] |
|  | Breast cancer [male] |
|  | Cancer of bladder |
|  | Cancer of bone and connective tissue |
|  | Cancer of brain |
|  | Cancer of brain and nervous system |
|  | Cancer of bronchus; lung |
|  | Cancer of connective tissue |
|  | Cancer of oesophagus |
|  | Cancer of eye |
|  | Cancer of hypopharynx |
|  | Cancer of intrathoracic organs |
|  | Cancer of kidney and renal pelvis |
|  | Cancer of larynx |
|  | Cancer of larynx, pharynx, nasal cavities |
|  | Cancer of lip |
|  | Cancer of liver and intrahepatic bile duct |
|  | Cancer of major salivary glands |
|  | Cancer of mouth |
|  | Cancer of nasopharynx |
|  | Cancer of nasal cavities |
|  | Cancer of oropharynx |
|  | Cancer of other endocrine glands |
|  | Cancer of other female genital organs |
|  | Cancer of other female genital organs (excluding uterus and ovary) |
|  | Cancer of other lymphoid, histiocytic tissue |
|  | Cancer of other male genital organs |
|  | Cancer of prostate |
|  | Cancer of stomach |
|  | Cancer of the gums |
|  | Cancer of the mouth floor |
|  | Cancer of tongue |
|  | Cancer of urinary organs (incl. kidney and bladder) |
|  | Cancer within the respiratory system |
|  | Cancer, suspected or other |
|  | Carcinoma in situ of skin |
|  | Cervical cancer |
|  | Cervical intraepithelial neoplasia [CIN] [Cervical dysplasia] |
|  | Chemotherapy |
|  | Colon cancer |
|  | Colorectal cancer |
|  | Hemangioma and lymphangioma, any site |
|  | Hemangioma of skin and subcutaneous tissue |
|  | Hodgkin's disease |
|  | Kaposi's sarcoma |
|  | Large cell lymphoma |
|  | Leukemia |
|  | Lymphoid leukemia |
|  | Lymphoid leukemia, acute |
|  | Lymphoid leukemia, chronic |
|  | Lymphosarcoma |
|  | Malignant neoplasm of bladder |
|  | Malignant neoplasm of female breast |
|  | Malignant neoplasm of gallbladder and extrahepatic bile ducts |
|  | Malignant neoplasm of head, face, and neck |
|  | Malignant neoplasm of kidney, except pelvis |
|  | Malignant neoplasm of liver, primary |
|  | Malignant neoplasm of other and ill-defined sites within the digestive organs and peritoneum |
|  | Malignant neoplasm of other urinary organs |
|  | Malignant neoplasm of ovary |
|  | Malignant neoplasm of ovary and other uterine adnexa |
|  | Malignant neoplasm of rectum, rectosigmoid junction, and anus |
|  | Malignant neoplasm of renal pelvis |
|  | Malignant neoplasm of retroperitoneum and peritoneum |
|  | Malignant neoplasm of small intestine, including duodenum |
|  | Malignant neoplasm of testis |
|  | Malignant neoplasm of unspecified male genital organ |
|  | Malignant neoplasm of uterus |
|  | Malignant neoplasm, other |
|  | Manlignant and unknown neoplasms of brain and nervous system |
|  | Melanomas of skin |
|  | Melanomas of skin, dx or hx |
|  | Monocytic leukemia |
|  | Multiple myeloma |
|  | Myeloid leukemia |
|  | Myeloid leukemia, acute |
|  | Myeloid leukemia, chronic |
|  | Myeloproliferative disease |
|  | Neoplasm of uncertain behavior |
|  | Neoplasm of uncertain behavior of breast |
|  | Neoplasm of uncertain behavior of male genital organs |
|  | Neoplasm of uncertain behavior of skin |
|  | Neoplasm of unspecified nature of digestive system |
|  | Neurofibromatosis |
|  | Nevus, non-neoplastic |
|  | Nodular lymphoma |
|  | Non-Hodgkins lymphoma |
|  | Other non-epithelial cancer of skin |
|  | Pancreatic cancer |
|  | Polycythemia vera |
|  | Reticulosarcoma |
|  | Secondary malignancy of bone |
|  | Secondary malignancy of brain/spine |
|  | Secondary malignancy of lymph nodes |
|  | Secondary malignancy of respiratory organs |
|  | Secondary malignant neoplasm |
|  | Secondary malignant neoplasm of digestive systems |
|  | Secondary malignant neoplasm of liver |
|  | Secondary malignant neoplasm of skin |
|  | Squamous cell carcinoma |
|  | Thyroid cancer |
| Neurological |  |
|  | Hemiplegia |
| Pregnancy Complications |  |
|  | Diabetes or abnormal glucose tolerance complicating pregnancy |
|  | Endocrine and metabolic disturbances of fetus and newborn |
|  | Hypertension complicating pregnancy, childbirth, and the puerperium |
|  | Other complications of pregnancy NEC |
|  | Preeclampsia and eclampsia |
| Respiratory |  |
|  | Asthma |
|  | Asthma with exacerbation |
|  | Chronic airway obstruction |
|  | Obstructive chronic bronchitis |
|  | Respiratory failure |
|  | Respiratory insufficiency |
|  | Wheezing |

# **Visualisation of the membership maps**

Figure S1(A) and (B) show the membership map generated by the GTM models trained on the UK Biobank and MIMIC-IV cohorts, respectively. These maps display the latent space containing a compressed representation of the entire original data space. Each point on the map represents a micro-cluster containing at least one participant, with the size of the point indicating the number of participants in the cluster: the larger the point, the more participants in the cluster and vice versa. Each participant has a probability of being assigned to every cluster but assignments below are the result of the participant being placed in the cluster with the highest probability.


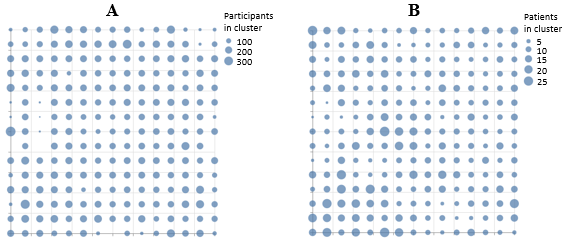


**Figure S1. Membership maps showing how participants/patients are distributed in the latent space**.

The size of each cluster reflect the number of participants/patients allocated to it. A) General population data from the UK Biobank. B) Critical care population data from the MIMIM-IV.

# **Characterisation of the AF phenotypes in the two cohorts**

The phenotypes were characterised by evaluating which variables showed a significant difference between clusters and identifying the distinct features.

## Phenotypes of the AF participants in the UK Biobank database

A breakdown of these features for each phenotype derived from the AF participants in the UK Biobank database is as follows:

Phenotype 1 (n =19,335)

The largest phenotype identified as part of the analysis, containing approximately 53% of the participants, shows the highest haematocrit percentage and mean corpuscular volume, as well as the highest levels of sodium in urine and direct bilirubin. This phenotype also contained the highest proportion of male participants (87.1%) which would be expected as the highest testosterone levels are seen in this phenotype. Additionally, this phenotype is categorised by participants that have the lowest pulse rate whilst having the highest peak expiratory flow rate and FEV1. This is also the most ethnically diverse phenotype, with it containing the height percentage of participants categorised as Black or Black British, Chinese, Mixed and Other ethnic group. It also features the highest alcohol consumption of the 5 phenotypes. A final distinguishing feature is that this phenotype has the highest levels of persistent AF across all phenotypes.

Phenotype 2 (n =4,480)

Characterised by having the youngest participant age group, participants in this phenotype were also the most likely to have chronic AF. Participants also showed the highest mean platelet distribution width, platelet distribution width, urate levels, albumin, alanine aminotransferase and gamma glutamyl transferase. Participants in this phenotype also showed a high, albeit not the highest, drinking levels with 2^nd^ highest saying they drink daily/almost daily and the least amount of people saying they drink infrequently at special occasions only.

Other key characteristics of this phenotype are the highest levels of triglycerides, lowest HDL cholesterol and SHBG levels. These features appear to be captured in the investigative variables with participants in this phenotype having the highest diastolic and systolic blood pressure, highest rates of type 1 and 2 diabetes and the highest rate of liver conditions.

Phenotype 3 (n =4,561)

Phenotype consists of the oldest participants whilst also being the most balanced concerning sex 51.4% being female. Participants in this phenotype are characterised by the highest values for neutrophil count and c-reactive protein and the lowest values for lymphocyte and monocyte percentages. They also have the highest levels of glycated haemoglobin and the highest urea levels. However, a higher level of urea is common in older people which may explain this reading. A key distinguishing factor is the participants have the highest hemiplegia levels, indicating that they are the most at risk of stroke.

The participants clustered in this phenotype also have the highest BMI and the lowest amount of weekly activity. The lowest alcohol intake levels are seen within the phenotype shown by having the highest percentage of participants that never drink or only drink on special occasions. The lowest number of white participants if seen in the phenotype, with it also having the highest percentage of Asian or Asian British participants. Regarding additional diseases, these participants are most likely to be diagnosed with additional comorbidities, with the phenotype showing the highest levels of circulatory system, endocrine/metabolic and respirator conditions. More specifically, the phenotype shows the highest rates of cardiovascular disease, peripheral vascular disease, hypertension, and kidney disease.

Phenotype 4 (n =47)

By far in the way the smallest phenotype, consisting of only a singular micro cluster. The participants here showed the lowest values for mean platelet volume, mean reticulocyte, and mean sphered cell volume whilst having the highest creatine value. Although unmentioned up until this point, this phecode has the most diverse genetic makeup, with 36 out of the 40 values being either a maximum or minimum value. This indicates that there may be some genetic difference in this between this phenotype when compared to the other four. These participants had the highest amount of weekly activity but the joint highest systolic blood pressure, matching that seen in phenotype 2. This is the only phenotype with no participants diagnosed with chronic AF participants, however they are the most likely to be diagnosed with a neoplasm condition, myocardial infarction, and coronary atherosclerosis.

Phenotype 5 (n =8,257)

This phenotype is defined as consisting almost entirely of white female participants (92% female participants with 99.4% being White or White British). Across the board, the participants in this phenotype show the lowest risk factors in every category in comparison to the other 5 phecodes. In addition to this, the participants also have the lowest BMI and lowest chance of having an additional comorbidity across all categories considered here. One feature that does stand out however is that participants in this phenotype are the most likely to be diagnosed with paroxysmal AF.

## Phenotypes of the AF patients in the MIMIC-IV database

Phenotype 1 (n = 1,705)

This is the largest phenotype identified out of the 4, consisting of 63% of patients. Patients in this phenotype are the youngest of the four, with a median age of 71. These patients also showed the highest anion gap, capillary refill, glucose, lactate, PEEP, phosphate, potassium, prothrombin time, and creatine, whilst also having the lowest haemoglobin, partial pressure of oxygen, oxygen saturation, systolic BP and joint lowest temperature with phenotype 3. The patients in this phenotype have the lowest GCS verbal response score, with highest rates of AKI.

Patients in this phenotype had the longest time to AF diagnosis once admitted to the ICY, as well as longest time spent in hospital and in the ICU. They also presented the highest rate mortality both in-hospital and after discharge, with the lowest time between mortality and ICU and hospital discharge.

Phenotype 2 (n = 631)

With regards to the variables used for modelling, patients in this phenotype had the least amount of stand out characteristics, with the expectations being that they presented the highest heart rate and respiratory rate and the joint lowest magnesium levels (shared with phenotype 4). Patients in this did however have the highest rate of non-invasive ventilation being used as well as the highest GCS scores for eye-opening and verbal response.

Phenotype 3 (n = 250)

This phenotype is defined by patients having the lowest values for anion gap, diastolic BP, glucose, PEEP, phosphate, platelet count, respiratory rate, creatine, and temperature, whilst also having the highest partial pressure of oxygen, pH, magnesium and fraction inspired oxygen. Outside of the modelling variables, patients were also the lowest on the GCS eye-opening and motor response scales and had the highest rates of invasive ventilation.

As opposed to phenotype 1, patients in this phenotype where diagnosed the quickest, and had the shortest length of stay both in-hospital and in the ICU. Furthermore, they had the lowest mortality rate both in-hospital and after discharge, with the longest time between discharge and mortality. Finally, patients here showed the lowest rates or ARDS.

Phenotype 4 (n = 109)

The final phenotype identified was the smallest of the 4, defined by contained the oldest patient’s cohort. The patients were also characterised with having the highest oxygen saturation, systolic and diastolic BP, temperature, haemoglobin, and platelet count. They also displayed the lowest fraction inspired oxygen, heart rate, lactate, magnesium, pH, phosphate, potassium, prothrombin time and creatine. Other defining features of this phenotype are the highest scores on GCS eye-opening and motor response scales, highest rates of ARDS and lowest rates of AKI and invasive ventilation.

# **Visualisation of the additional investigative variables**

## Visualisation of the membership map stratified by sex


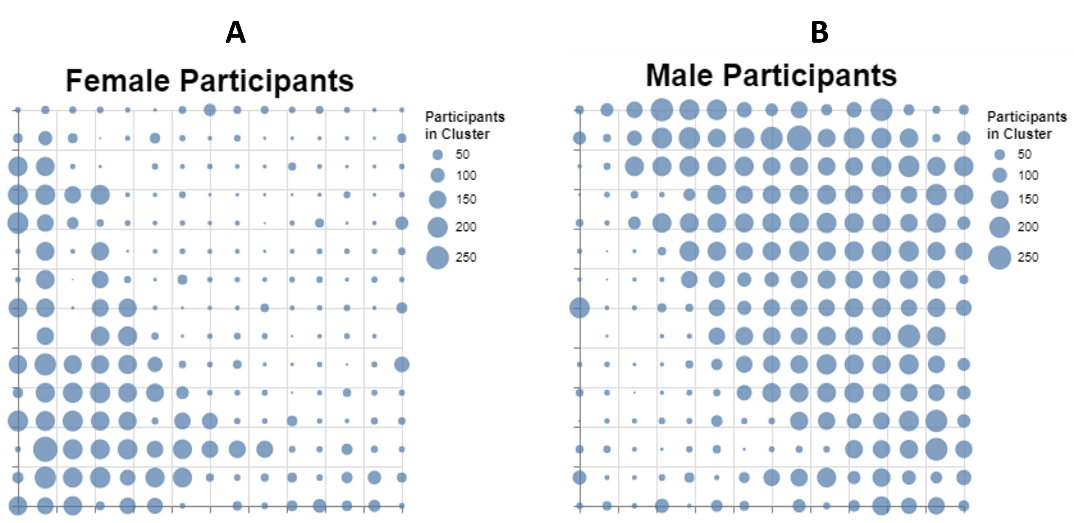


**Figure S2. Membership map generated by GTM stratified by the sex of the participant.** A) Only female participants; B) Only male participants.

## UK Biobank Investigative Variables

**
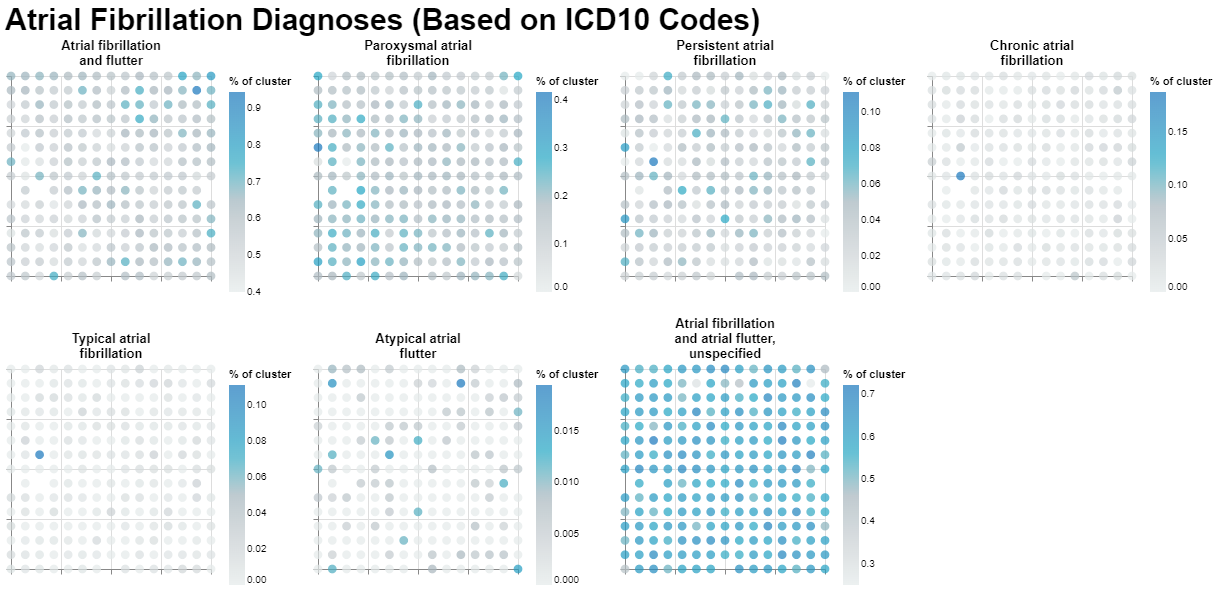
**

**
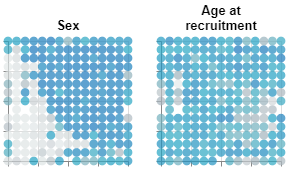
** **
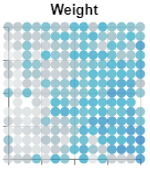
**

**
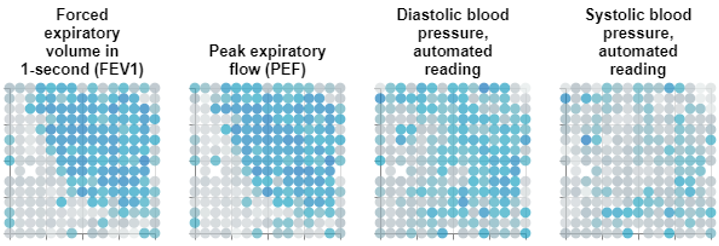
**


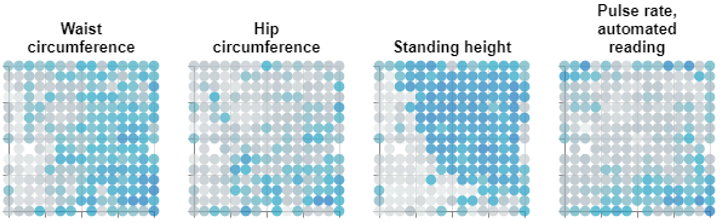


**
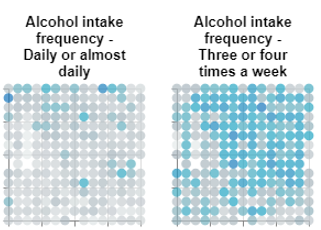

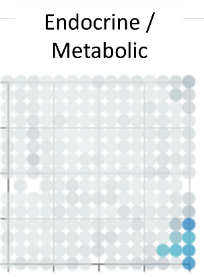

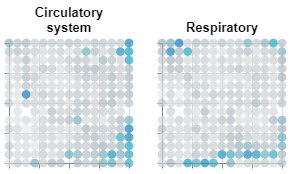
**

**
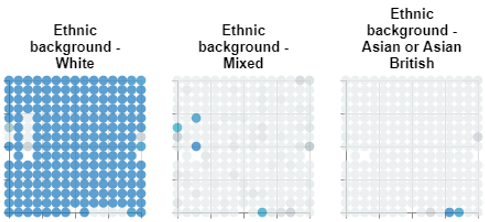

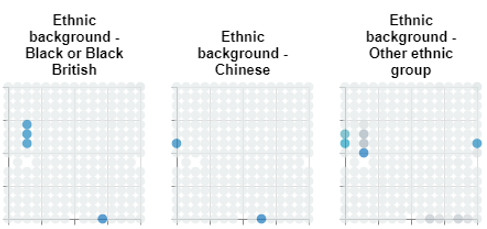

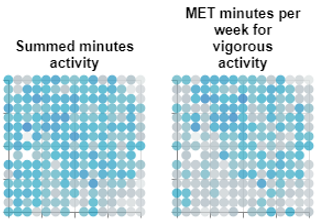

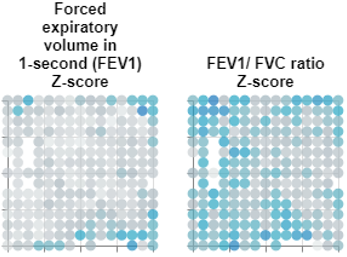
**

**
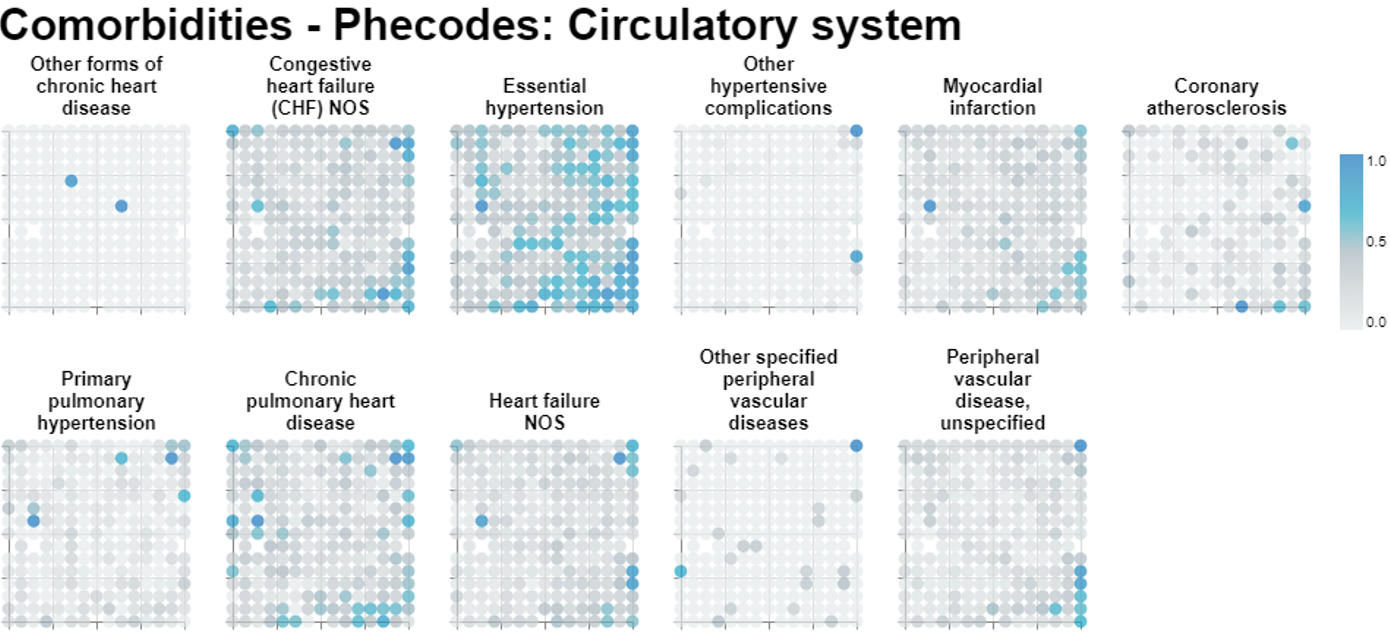
**

**
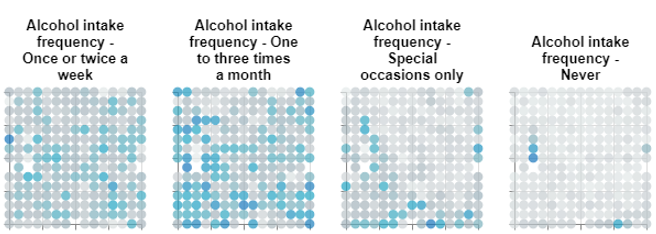
**

**
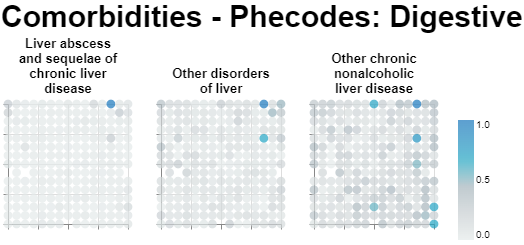
**

**
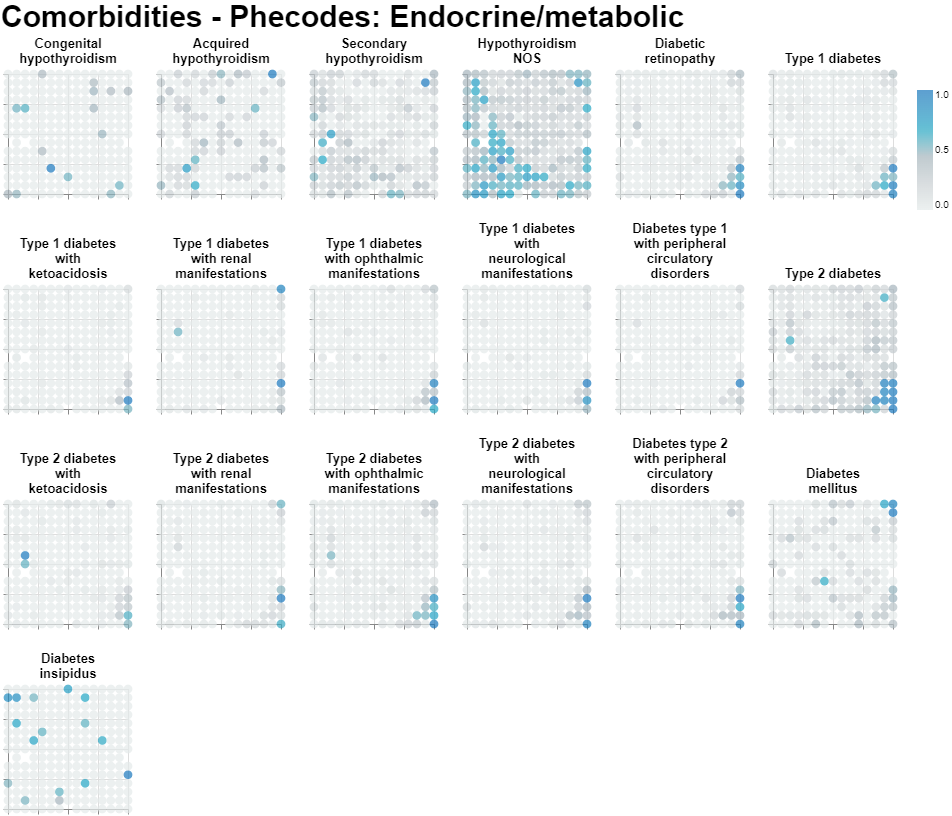
**

**
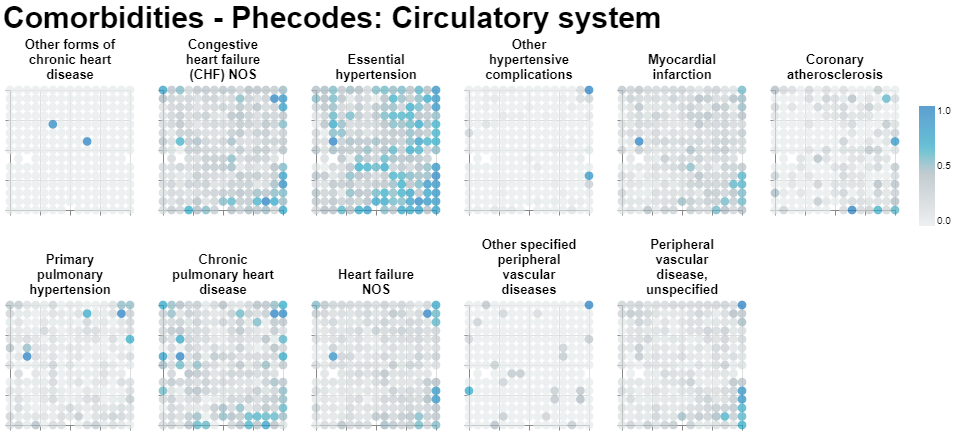
**

**
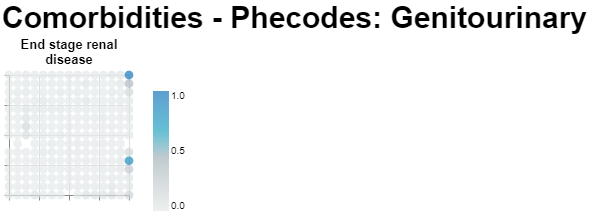
**

**
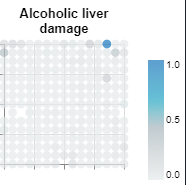
**


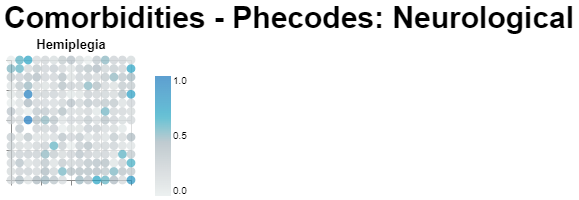


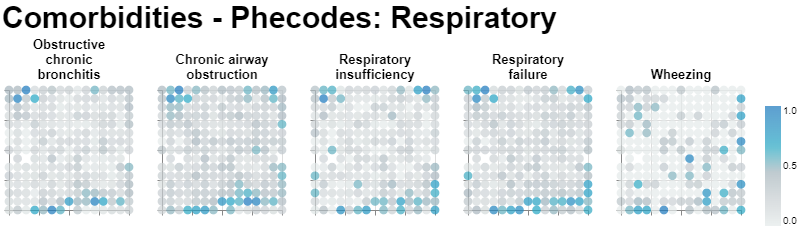


## MIMIC-IV Investigative Variables


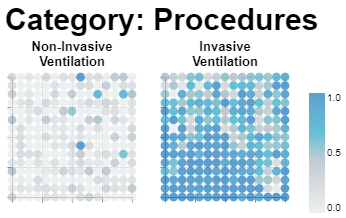


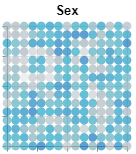

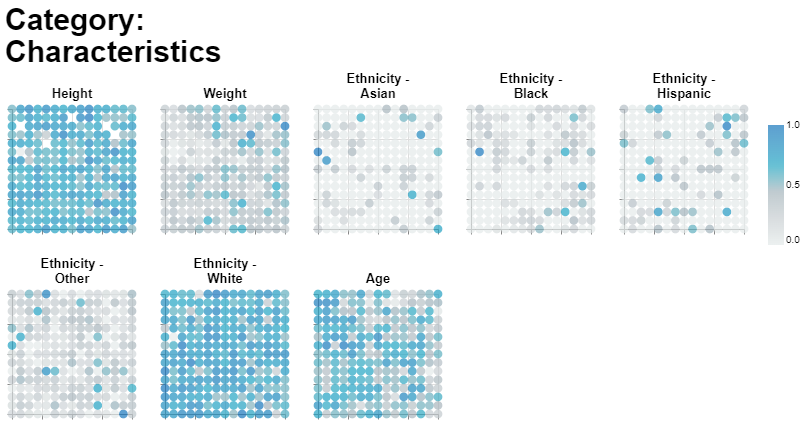


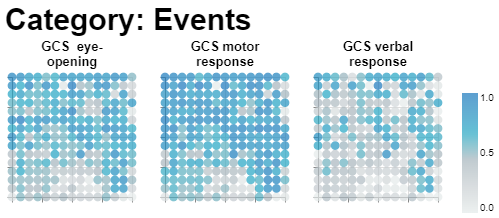


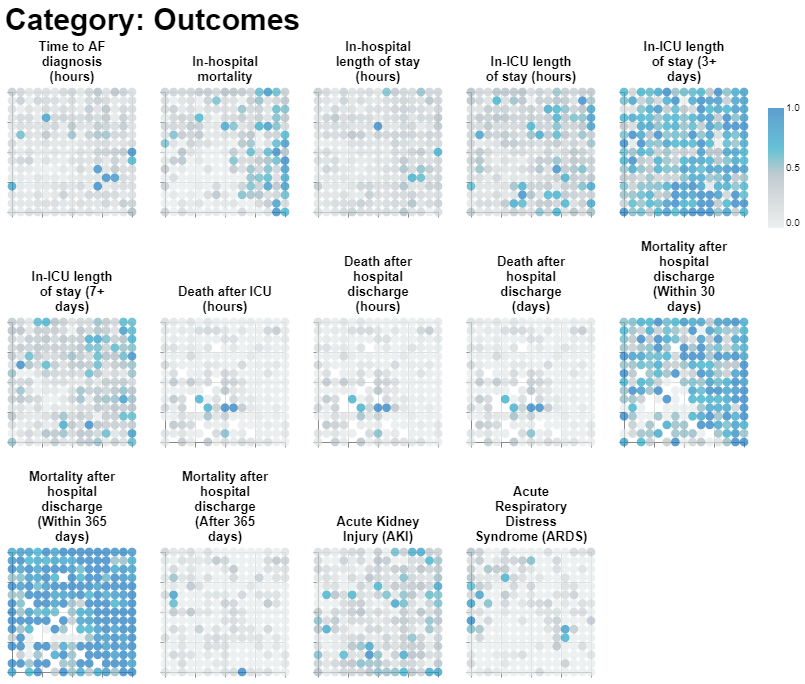


# **References**

1 Bishop CM, Svensén M, Williams CKI. GTM: The Generative Topographic Mapping. *Neural Comput* 1998; **10**: 215–34.

2 Kohonen T. Self-Organizing Maps. Berlin, Heidelberg: Springer Berlin Heidelberg, 2001 DOI:10.1007/978-3-642-56927-2.

3 Bishop C, Svensén M, Williams C. GTM: A Principled Alternative to the Self-Organizing Map. In: Mozer MC, Jordan M, Petsche T, eds. Advances in Neural Information Processing Systems. MIT Press, 1996.

4 Van Der Maaten L, Hinton G. Visualizing data using t-SNE. *Journal of Machine Learning Research* 2008; **9**: 2579–625.

5 McInnes L, Healy J, Saul N, Großberger L. UMAP: Uniform Manifold Approximation and Projection. *J Open Source Softw* 2018; **3**: 861.

6 van Buuren S, Groothuis-Oudshoorn K. mice: Multivariate Imputation by Chained Equations in R. *J Stat Softw* 2011; **45**: 1–67.

7 Romiti GF, Proietti M, Bonini N, *et al.* Clinical Complexity Domains, Anticoagulation, and Outcomes in Patients with Atrial Fibrillation: A Report from the GLORIA-AF Registry Phase II and III. *Thromb Haemost* 2022. DOI:10.1055/S-0042-1756355.

8 Lip GYH, Genaidy A, Tran G, Marroquin P, Estes C, Sloop S. Improving Stroke Risk Prediction in the General Population: A Comparative Assessment of Common Clinical Rules, a New Multimorbid Index, and Machine-Learning-Based Algorithms. *Thromb Haemost* 2021; **122**. DOI:10.1055/a-1467-2993.

9 Allan V, Honarbakhsh S, Casas JP, *et al.* Are cardiovascular risk factors also associated with the incidence of atrial fibrillation?: A systematic review and field synopsis of 23 factors in 32 population-based cohorts of 20 million participants. Thromb Haemost. 2017; **117**. DOI:10.1160/TH16-11-0825.

10 Nso N, Bookani KR, Metzl M, Radparvar F. Role of inflammation in atrial fibrillation: A comprehensive review of current knowledge. *J Arrhythm* 2021; **37**: 1–10.

11 Mohanty S, Hall A, Mohanty P, *et al.* Being Asymptomatic With Atrial Fibrillation: Is It a Genetic Trait? *J Am Coll Cardiol* 2016; **67**: 677.

12 Kalarus Z, Mairesse GH, Sokal A, *et al.* Searching for atrial fibrillation: looking harder, looking longer, and in increasingly sophisticated ways. An EHRA position paper. Europace. 2023; **25**. DOI:10.1093/europace/euac144.

13 Alonso A, Krijthe BP, Aspelund T, *et al.* Simple Risk Model Predicts Incidence of Atrial Fibrillation in a Racially and Geographically Diverse Population: the CHARGE‐AF Consortium. *J Am Heart Assoc* 2013; **2**. DOI:10.1161/JAHA.112.000102.

14 Lip GYH, Skjøth F, Nielsen PB, Larsen TB. Evaluation of the C2HEST Risk Score as a Possible Opportunistic Screening Tool for Incident Atrial Fibrillation in a Healthy Population (From a Nationwide Danish Cohort Study). *American Journal of Cardiology* 2020; **125**. DOI:10.1016/j.amjcard.2019.09.034.

15 Johnston BW, Chean CS, Duarte R, *et al.* Management of new onset atrial fibrillation in critically unwell adult patients: a systematic review and narrative synthesis. *Br J Anaesth* 2022; **128**: 759–71.

16 Bosch NA, Cimini J, Walkey AJ. Atrial Fibrillation in the ICU. *Chest* 2018; **154**: 1424–34.

17 O’Driscoll BR, Smith R. Oxygen Use in Critical Illness. *Respir Care* 2019; **64**: 1293–307.

18 Papadopoulou A, Harding D, Slabaugh G, Marouli E, Deloukas P. Prediction of atrial fibrillation and stroke using machine learning models in UK Biobank. *medRxiv* 2022.

19 Wu P, Gifford A, Meng X, *et al.* Mapping ICD-10 and ICD-10-CM Codes to phecodes: Workflow development and initial evaluation. *JMIR Med Inform* 2019; **7**: 1–13.
